# Mitigating Solidification Cracking in LPBF-Processed K418 Superalloy via Substrate Preheating and Layer Thickness Optimization

**DOI:** 10.3390/ma19030501

**Published:** 2026-01-27

**Authors:** Deqin Zhao, Jie Pei, Chenxue Ma, Rengeng Li

**Affiliations:** Key Laboratory for Light-Weight Materials, Nanjing Tech University, Nanjing 210009, China; 202261203204@njtech.edu.cn (D.Z.); peijie@njtech.edu.cn (J.P.); machenxue@njtech.edu.cn (C.M.)

**Keywords:** laser powder bed fusion, nickel-based superalloy, cracks, additive manufacturing

## Abstract

This study systematically investigates the influence of key process parameters—layer thickness and substrate preheating—on solidification cracking in K418 nickel-based superalloy fabricated by laser powder bed fusion (LPBF). For a 30 μm layer, preheating to 350 °C combined with a volumetric energy density (VED) of 60–80 J/mm^3^ effectively suppressed hot cracking while achieving a relative density > 99%. Preheating to 200 °C showed limited effectiveness. Without preheating, increasing the layer thickness to 60 μm reduced cracking compared to 30 μm, yet preheating became counterproductive under this thicker condition due to excessive thermal accumulation and increased shrinkage stress. Microscopic analysis revealed that cracks propagated along high-angle grain boundaries accompanied by the segregation of low-melting-point elements (O, B, Si, C), with cracking attributed to thermal stress and grain boundary weakening during rapid solidification. This work establishes 350 °C preheating with moderate VED as an effective strategy for manufacturing high-density, crack-minimized K418 alloy components via LPBF.

## 1. Introduction

Nickel-based high-temperature alloys, thanks to their excellent high-temperature strength, oxidation and thermal corrosion resistance, and good creep and fatigue resistance, have become key materials for hot-end components of high-end equipment such as aircraft engines and gas turbines (e.g., turbine blades, guide vanes, and turbine disks) [1]. Currently, K418 alloy components are mainly produced using the investment casting process to meet the formation requirements of their complex internal cooling channels. While investment casting can form complex shapes, it still has significant limitations when dealing with high-alloy, wide solidification range alloys like K418. To overcome the limitations of traditional casting, additive manufacturing technology has shown significant advantages, particularly laser powder bed fusion (LPBF), which has demonstrated considerable potential [2,3]. This technology provides a new approach for producing the K418 alloy: first, it offers high design freedom, enabling the creation of lightweight and functionally integrated components with complex internal flow channels and lattice structures that are difficult to achieve with traditional processes [4,5]; second, its extremely rapid cooling rates (typically 10^3^–10^6^ K/s) help form fine cellular/dendritic structures, effectively alleviating element segregation and improving composition uniformity; third, the near-net shape formation characteristic helps improve material utilization, which is particularly suitable for these expensive high-temperature alloys [6,7]. However, the LPBF fabrication of K418 also faces severe challenges, mainly manifested as high sensitivity to solidification cracking, resulting in inadequate microstructure and performance. The root cause of cracking lies in the mismatch between the wide solidification temperature range and element segregation tendency of the K418 alloy and the extremely high thermal stress and rapid thermal cycling in the SLM process, which easily leads to the formation of low-melting liquid films between dendrites and triggers intergranular cracks [8,9].

Currently, researchers have conducted specific studies on this issue, mainly focusing on process control to address cracking problems. This includes research on laser parameter optimization, substrate preheating, and the effect of layer thickness on cracking [10,11]. In terms of laser parameter optimization, researchers systematically altered power, speed, and scanning strategies and conducted a detailed analysis of density and crack density. They found that there is an optimal energy window that can improve density but cannot completely eliminate cracks; scanning strategies can distribute stress, but their effect is limited. Regarding preheating, researchers studied the effect of different preheating temperatures (from room temperature to 1000 °C) on IN713LC cracking behavior and found that substrate preheating is one of the most effective ways to suppress cracks. High-temperature preheating (>800 °C) is key to achieving crack-free formation, significantly reducing thermal stress and the tendency for solidification cracks [12]. Concerning layer thickness, researchers compared the cracking behavior of IN713LC at different layer thicknesses and found that layer thickness is not an independent variable; its effect is closely related to preheating temperature. Without sufficient preheating, merely adjusting layer thickness has very little effect on eliminating cracks [13,14,15].

This study mainly examined the effects of laser parameters (laser power and scanning speed), layer thickness, and preheating temperature on the formation quality of K418 alloy and conducted a detailed investigation into the relationship between process parameters and the formation of solidification cracks. At the same time, the characteristics of solidification cracks were analyzed in detail through EBSD and EDS, clarifying the mechanism of solidification cracking in K418 alloy.

## 2. Materials and Methods

### 2.1. Experimental Materials

**Figure 1:**
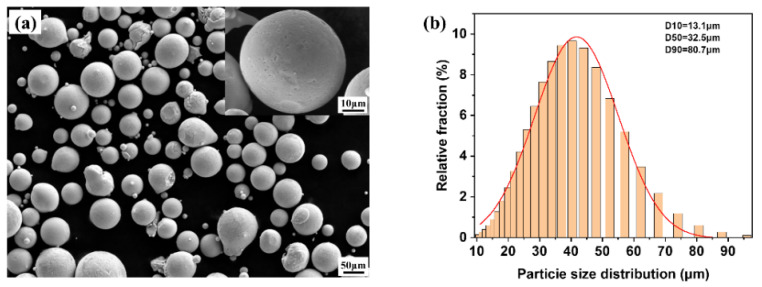
(**a**) SEM images showing the particle morphology of the gas-atomized K418 powder; (**b**) particle size distribution.

The K418 high-temperature alloy powder used in this study was supplied by Xi’an Bright Laser Additive Manufacturing Technology Co., Ltd. (Xi’an, China). Its main chemical composition and mass fractions are shown in Table 1. This alloy contains relatively high amounts of γ’ phase-forming elements such as Al, Ti, and Nb [16,17,18]. The microstructure of K418 powder prepared by gas atomization is shown in Figure 1a; the powder particles are generally nearly spherical, with a small portion having some fine satellite particles. The particle size distribution is shown in Figure 1b, with a powder size range of 15–53 μm and an average particle size of 32.5 μm. The powder exhibits good flowability and filling properties, making it suitable for the selective laser melting (SLM) process.

### 2.2. Preparation of Metallographic Samples and Tensile Specimens by SLM

In order to study the effects of printing process parameters on the formation quality of K418, 10 × 10 × 10 mm^3^ cube samples were prepared using different process parameters. This experiment primarily investigates the effects of laser parameters (laser power and scanning speed) on cracks, porosity, and lack-of-fusion metallurgical defects under a scanning strategy with a 67° interlayer rotation. At the same time, wide-range laser parameter printing experiments were conducted with layer thicknesses of 30 μm and 60 μm to examine the formation window of K418 alloy at different layer thicknesses. In addition, substrate preheating is considered to effectively reduce the temperature gradient, decrease residual stress during printing, and thus lower the risk of cracking. Therefore, while studying layer thickness, the formation window of K148 alloy under three conditions—no substrate preheating, substrate preheated to 200 °C, and substrate preheated to 350 °C—was compared and analyzed, with particular attention to its effect on crack formation. The printing parameters used in this experiment are shown in Table 2.

**Figure 2:**
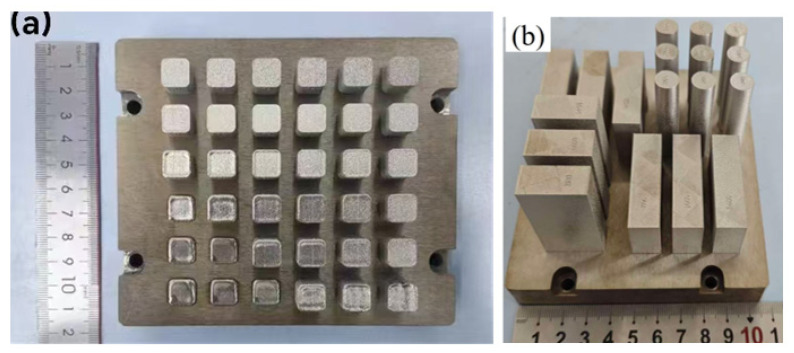
The printed physical diagram. (**a**) Metallographic specimen; (**b**) Tensile specimen.

In addition, to study the effect of process parameters on the mechanical properties of K418, a block 3D model of the component was first created using Magics software (24.01) before printing. Under the conditions of a layer thickness of 30 μm, a power of 200 W, a scan spacing of 0.1 mm, and a checkerboard 67° rotation scanning strategy, three different scanning speeds of 800, 1000, and 1400 mm/s were selected, along with no preheating and preheating to 350 °C to investigate the influence of process parameters on the mechanical properties of K418. The printed samples are shown in Figure 2.

### 2.3. Material Characterization Methods

**Figure 3:**
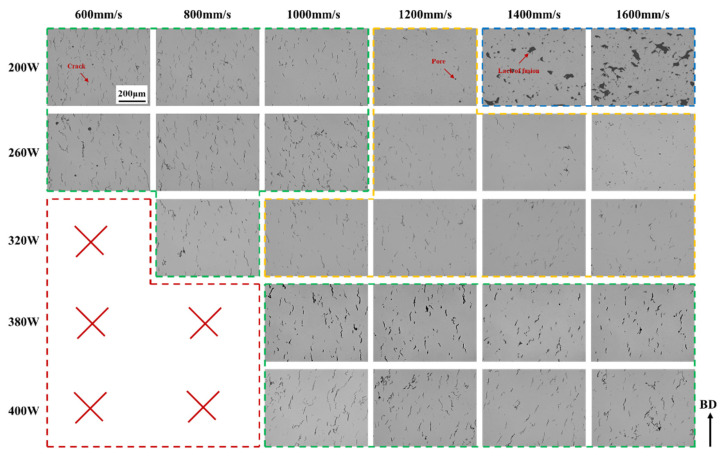
OM images of K418 samples with 60 μm layer thickness processed by SLM at different scanning rates and different powers.

After SLM formation, the printed component is separated from the substrate, and the sample surface is progressively ground with SiC sandpaper of different grits. At the same time, the ground component is placed on a polishing machine at a speed of 500 rpm for polishing until the surface shows no obvious scratches and exhibits a mirror-like shine. The polished sample is then placed on the stage of a Axio Vert.A1 (Zeiss, Jena, Germany) inverted optical microscope to observe and analyze the distribution of surface defects and cracks on each sample. As shown in Figure 3, six different OM images were obtained from different cross-sectional areas of the sample, and the crack density was recorded and analyzed using Image J software (http://imagej.org, accessed on 7 June 2025). Cracks and pores can be distinguished based on circularity C [17]: C = 4π(A/P^2^) (where A represents the defect area and P represents the defect perimeter). When the circularity C value approaches 1, the defect shape is closer to a perfect circle. Defects with a circularity value below 0.3 are classified as cracks, while those exceeding 0.3 are determined to be pores.

Subsequently, the polished surface of the sample was chemically etched using aqua regia in a volume ratio of 3:1 (hydrochloric acid to nitric acid) for 25 s. After etching, the sample surface was observed again using an inverted optical microscope to further analyze changes and characteristics in its microstructure [19].

In this study, a tungsten filament scanning electron microscope (SEM, TESCAN CLARA, Brno, Czech Republic) was used to observe and record the microstructural evolution of the transverse and longitudinal sections of samples under different process parameters, the distribution characteristics of the reinforcing phases, and the morphology of tensile fracture surfaces. Additionally, combined with electron backscattered diffraction (EBSD) analysis, the obtained Kikuchi patterns were compared with a database to accurately identify the crystal structure, orientation, and other microstructural information of the samples. During the EBSD analysis, the backscattered electron (BSE) imaging mode was used, with specific operational parameters set as follows: an accelerating voltage of 20 kV, beam current of 15 nA, and a working distance of 10 mm. The acquired EBSD data were subsequently processed and analyzed using OIM (Orientation Imaging Microscopy) software (6 × 64). During scanning, the step size was set to 0.7 μm to obtain high-resolution data.

## 3. Result and Discussion

### 3.1. Effect of Layer Thickness on Cracking of K418 High-Temperature Alloy

An optical microscopy examination of the central region of the K418 samples fabricated with a 60 μm layer thickness (Figure 3) revealed distinct defect characteristics across different laser power ranges. At lower power levels (140–200 W), the predominant defects were unmelted zones and occasional pores. This is attributed to insufficient energy input to fully melt the thicker powder bed; however, the increased layer thickness effectively lowers the volumetric energy density, thereby mitigating the high thermal stresses typical of the 30 μm condition and reducing crack susceptibility. Within the medium-to-high power range (320–380 W), pore formation became more pronounced. Notably, compared to the 30 μm reference, the 60 μm layers exhibited a significant reduction in solidification cracking, although some unmelted regions and pores persisted. These observations suggest that the thicker layer provides a broader process window, enabling a compromise between unmelted defects and porosity through a careful adjustment of power and scanning speed while effectively avoiding crack formation. The requirement for higher energy input to melt the increased powder volume shifts the optimal processing window toward either lower scan speeds or higher laser power for the 60 μm condition.

**Figure 4:**
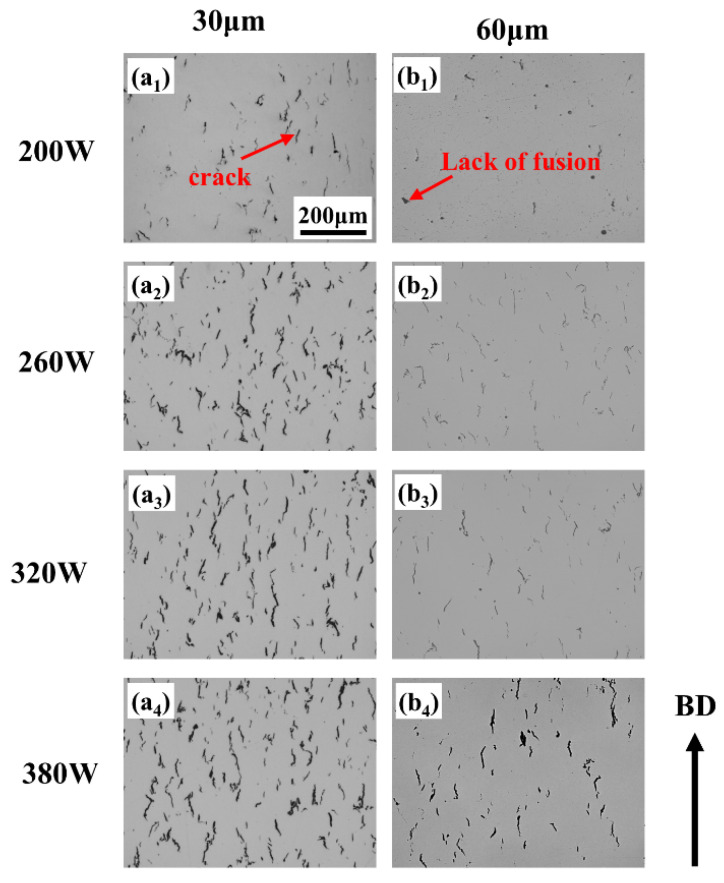
OM images of samples with different layer thicknesses processed by SLM at a scanning rate of 1200 m/s. (**a_1_**–**a_4_**) 30 μm; (**b_1_**–**b_4_**) 60 μm.

Figure 4 presents OM images of SLM-processed samples with 30 μm and 60 μm layer thicknesses at a fixed scanning speed of 1200 mm/s. For the 30 μm layer (Figure 4(a_1_)), numerous irregular lack-of-fusion defects are observed, resulting from severely insufficient energy input that prevents continuous melt pool formation and adequate remelting of the underlying material. As the power increases (Figure 4(a_2_)), lack-of-fusion defects decrease, but pronounced macroscopic cracks appear along grain boundaries, indicating that while melting is improved, high cooling rates and associated thermal stress induce cracking. With further power elevation (Figure 4(a_2_–a_4_)), the microstructure becomes progressively denser and lack-of-fusion defects diminish; however, a dense network of microcracks develops, demonstrating that higher power enhances densification yet cannot alleviate the high thermal stresses generated during rapid solidification. For the 60 μm layer under low power (Figure 4(b_1_)), lack-of-fusion defects are still present, yet microcracks are relatively scarce. In the power range of 200–260 W, microcrack density remains low and the microstructure is notably denser compared to the 30 μm condition. When power exceeds 320 W, distinct microcracks emerge, predominantly aligned along the build direction (BD).

**Figure 5:**
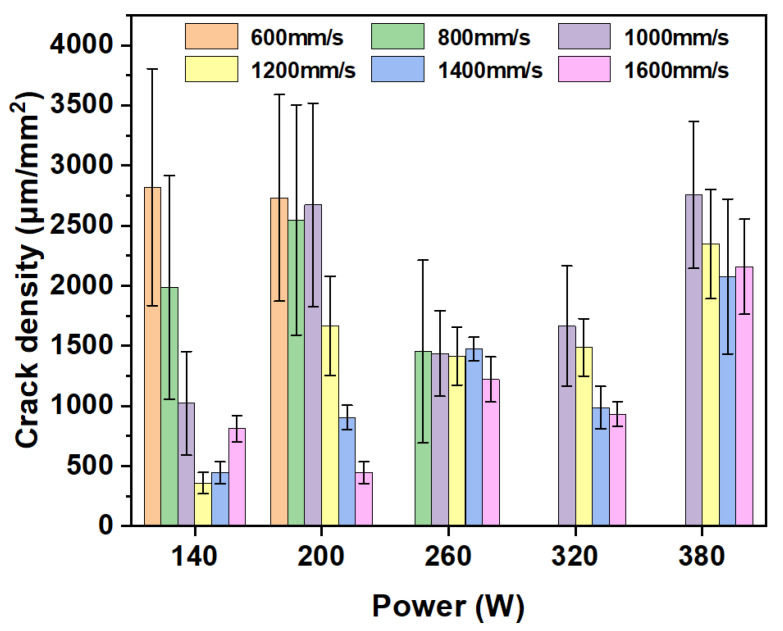
The crack density statistics of 60 μm layer thickness samples processed by SLM.

**Figure 6:**
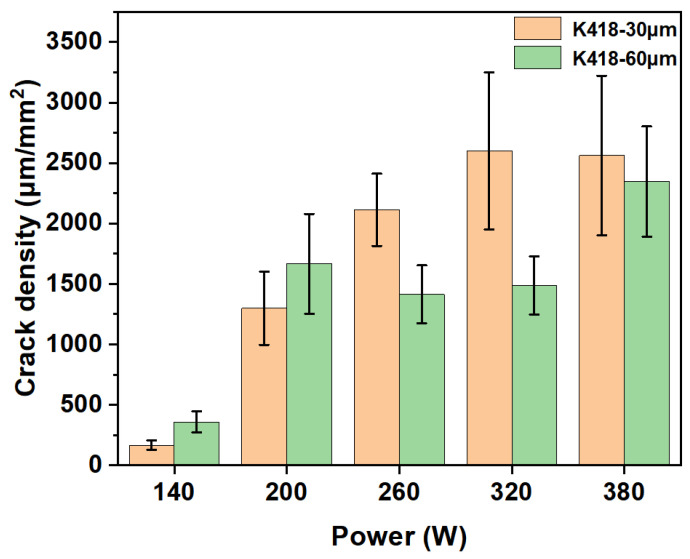
The crack density statistics of different layer thickness samples with a scanning rate of 1200 mm/s processed by SLM.

A quantitative analysis of crack density in the 60 μm layer samples is presented in Figure 5. As illustrated in Figure 6, crack density exhibits a marked monotonic increase with rising laser power at a given scanning speed—a trend consistent with that observed for the 30 μm layer. For any fixed power level, lower scanning speeds (e.g., 600 or 800 mm/s) yield reduced crack density, whereas higher speeds (e.g., 1400 or 1600 mm/s) lead to greater crack density. For instance, at 140 W, crack density measures 358.02 μm/mm^2^ at 600 mm/s but rises sharply to 2818.78 μm/mm^2^ at 1200 mm/s. The combination of high power and low speed (e.g., 380 W/600 mm/s) corresponds to high energy input, which promotes the formation of a larger, more stable melt pool with improved fluidity, thereby facilitating the backfilling of shrinkage cavities between dendrites. Overall, crack densities for the 60 μm samples are generally lower than those of their 30 μm counterparts under identical parameters, and the crack density distribution appears more gradual. Notably, a distinct region of low crack density emerges around 260 W, underscoring that the 60 μm layer offers a broader and more stable processing window. The increased layer thickness effectively acts as an extended thermal buffer, elevating heat capacity and attenuating heat transfer efficiency, which significantly curbs the cooling rate and enhances thermal accumulation. This mechanism delivers two key advantages: first, it alleviates thermal stresses and temperature gradients; second, it allows extended time for elemental diffusion, thereby mitigating detrimental segregation. Together, these factors substantially inhibit both crack initiation and propagation [20,21,22].

### 3.2. Effect of Substrate Preheating on Cracking of K418 Superalloy

**Figure 7:**
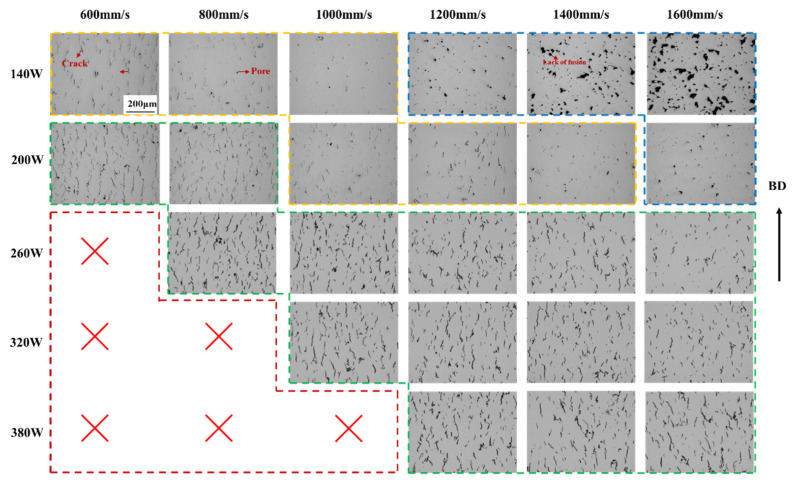
OM images of K418 samples with 30 μm layer thickness processed by SLM at different scanning rates and different powers.

**Figure 8:**
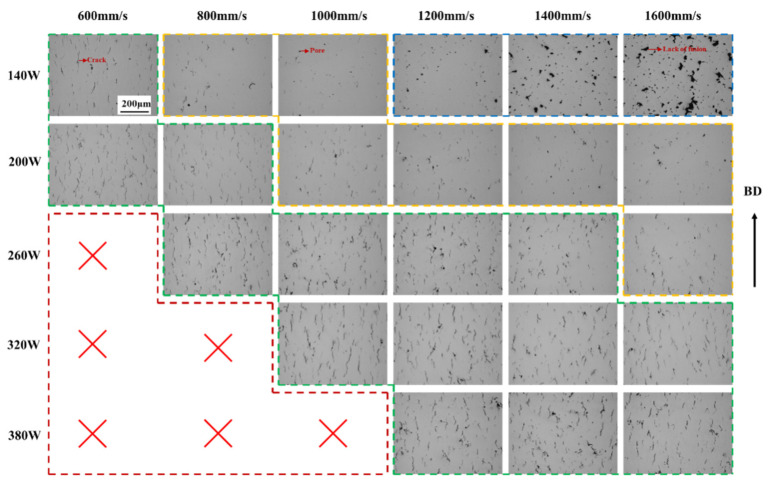
OM images of K418 samples with 30 μm layer thickness preheated at 200 °C, different scanning rates, and different powers processed by SLM.

**Figure 9:**
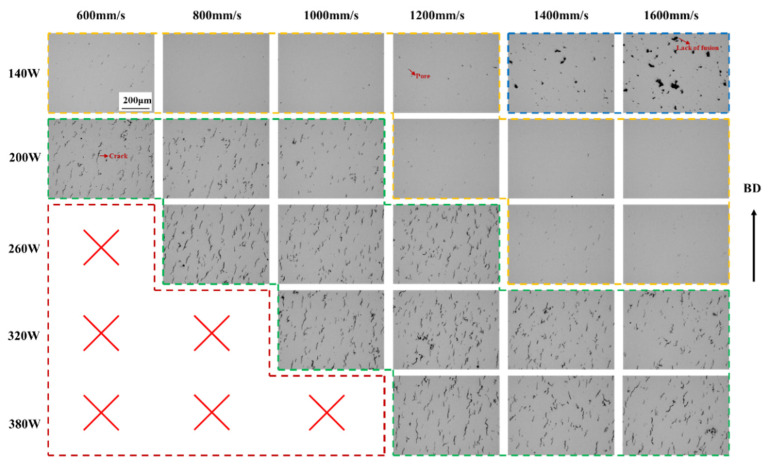
OM images of K418 samples with 30 μm layer thickness preheated at 350 °C, different scanning rates, and different powers processed by SLM.

Based on the above research, the effect of substrate preheating on the cracking of K418 superalloy is also explored. The central position of the K418 sample at different preheating temperatures (200 °C and 350 °C) under SLM-processed 30 μm layer thickness was observed using optical microscopy, as shown in Figure 7 and Figure 8. Comparing the samples without preheating the substrate and preheating the substrate at 200 °C and 350 °C, it can be found that the preheating of the substrate leads to a widening of the process window and a significant improvement in the forming quality. Compared with the condition of no preheating of the substrate, 200 °C preheating can effectively reduce the risk of cracking and the crack density is reduced, indicating that 200 °C preheating effectively reduces the cooling rate and thermal stress. After the crack is suppressed, pores become a major defect of concern, appearing in the medium scan speed region (e.g., 1000–1200 mm/s). The non-fusion defect at low power (140 W) remains but to a lesser extent. The print failure parameters in the high-power region (320 W, 380 W) are reduced, indicating that preheating makes high-power parameters feasible. Preheating at 200 °C effectively reduced the crack density and broadened the process window, but there were still some microscopic cracks. To this end, the substrate preheating temperature was further increased to 350 °C, and the results are shown in Figure 9. Under the condition of 30 μm layer thickness, the process window of preheating at 350 °C was significantly widened, and better formation quality was obtained. Compared with 200 °C preheating, the crack density is significantly reduced, no obvious cracking phenomenon is found in the moderate power and scanning rate range, and the pore defects are significantly reduced. This indicates that the higher preheating temperature provides a more stable thermal environment, which is conducive to smooth solidification of the melt pool and gas escape. Non-fusion at low power (140 W) is still an inherent phenomenon due to insufficient energy input, but it is reduced compared to no warm-up and 200 °C preheating. A uniform, dense, defect-free microstructure is obtained over a wider range of parameters, especially 200–380 W.

The preheating temperature is one of the most effective and critical process measures to suppress solidification cracks in K418 alloy SLM. The mechanism lies in the fact that preheating significantly reduces the cooling rate and temperature gradient of the melt pool, thereby greatly reducing residual thermal stress and preventing the material from cracking in the brittle temperature range. At the same time, preheating helps element diffusion and alleviates harmful grain boundary segregation. Preheating at 200 °C combined with appropriate laser parameters can effectively reduce the risk of cracking caused by thermal stress, but it may not be sufficient to fully optimize melt pool dynamics. Preheating at 350 °C provides better thermal input, reduces temperature gradients and thermal stress, and significantly decreases the formation of solidification cracks in the sample. In addition, a higher preheating temperature can promote melt pool stability and gas escape while significantly reducing porosity to achieve high-density samples. The main issue without preheating is cracks caused by high stress. With preheating at 200 °C, the main defect shifts to porosity are caused by melt pool instability. Preheating at 350 °C effectively resolves both cracking and porosity issues, allowing the process optimization focus to shift towards controlling the microstructure and properties.

**Figure 10:**
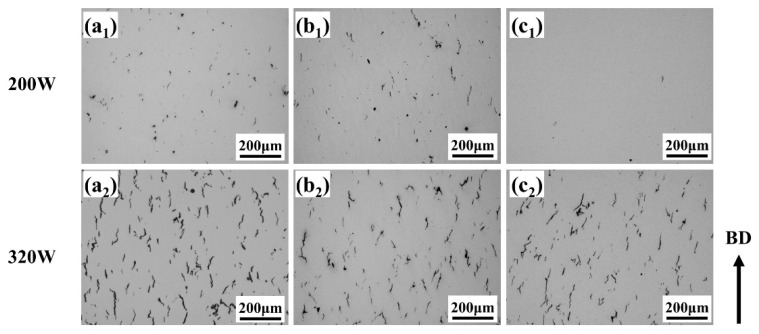
The OM diagram of the 30 μm preheating condition of SLM processing: (**a_1_**,**a_2_**) no substrate preheating; (**b_1_**,**b_2_**) substrate preheating at 200 °C; (**c_1_**,**c_2_**) substrate preheating at 350 °C.

Figure 10 shows OM images of SLM-processed samples with a layer thickness of 30 μm under preheating conditions. It can be seen that at 200 W without preheating, typical lack-of-fusion defects and solidification cracks are present in the microstructure. Preheating to 200 °C significantly reduces the lack of fusion defects, while preheating to 350 °C almost eliminates them, resulting in a uniform and dense microstructure. As for 320 W, there is no significant change. This is because the increased power raises the energy input, promoting melting, but the extremely high cooling rate and thermal stresses still exist, potentially causing keyhole effects (leading to porosity) and thermal stress cracking. The above results indicate that substrate preheating needs to be combined with appropriate laser power and scanning speed. Under conditions of relatively high energy input, substrate preheating alone still encounters difficulty in effectively suppress cracking.

**Figure 11:**
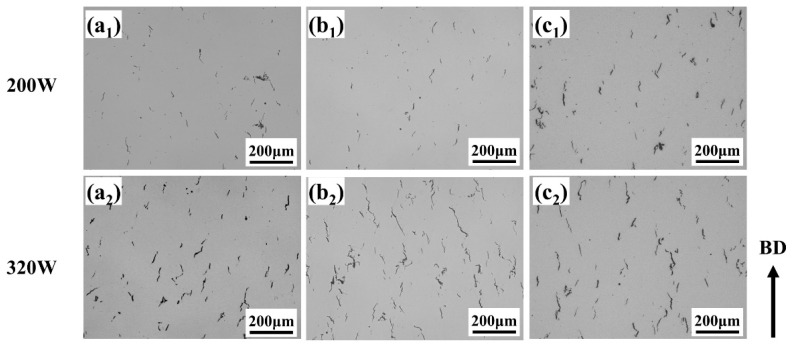
OM diagram of 60 μm preheating processed by SLM: (**a_1_**,**a_2_**) without substrate preheating; (**b_1_**,**b_2_**) substrate preheating at 200 °C; (**c_1_**,**c_2_**) substrate preheating at 350 °C.

Figure 11 shows the optical microscope (OM) image of a 60 μm layer thickness preheating condition in SLM processing. By comparing with Figure 11, it can be seen that under the 30 μm layer thickness condition, the extremely high cooling rate generates significant thermal stress, leading to severe solidification cracks. The primary function of preheating is to reduce the cooling rate and stress to suppress cracking. When the layer thickness is increased to 60 μm, however, substrate preheating instead exacerbates crack formation, which is contrary to the result observed at a 30 μm layer thickness. The above differences indicate that when the layer thickness is larger, although preheating can lower the temperature gradient and thus reduce thermal stress, the increase in layer thickness along with high preheating temperature significantly increases material melting, enlarging the molten pool volume. The shrinkage of the melt causes a marked increase in stress, and due to the larger molten pool, the solidification time is prolonged, increasing the risk of solidification cracking under stress.

**Figure 12:**
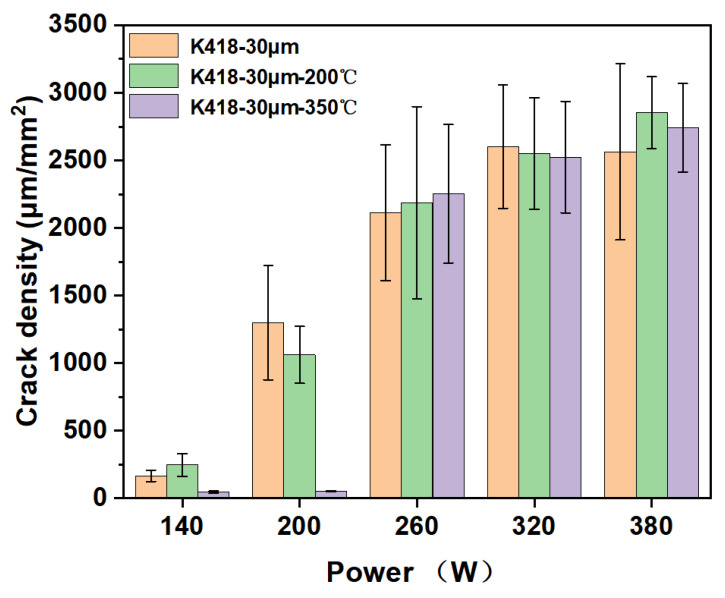
The crack density statistics of whether the substrate is preheated by SLM processing.

Figure 12 shows the statistical diagram of crack density for SLM-processed substrates with a 30 μm layer thickness, comparing whether the substrate was preheated or not. It can be seen that under all preheating conditions, the crack density increases significantly with the increase in laser power. In the low-power range (140–200 W), the crack density remains at a relatively low level, about 500–1000 μm/mm^2^. At this time, the energy input is low, the melt pool is small, and the resulting thermal stress is relatively controllable. In the medium-power range (260–320 W), the crack density enters a rapid growth phase, increasing from about 1500 μm/mm^2^ to 2000 μm/mm^2^. The increased energy input causes the melt pool to expand, and the thermal and shrinkage stresses after cooling are significantly intensified. In the high-power range (380 W), the crack density reaches its peak, about 2500 μm/mm^2^. High energy input causes intense thermal behavior; while it aids powder melting, it also generates maximum thermal stress, leading to rapid crack initiation and propagation. For K418 alloy with a 30 μm layer thickness, laser power is one of the main driving factors for crack formation. Excessively high working power will directly worsen the crack density. When preheated to 200 °C, the crack density curve over the entire power range almost completely overlaps with that of the non-preheated condition. This indicates that 200 °C substrate preheating has limited effect on suppressing cracks in 30 μm layer K418 alloy. When the substrate preheating temperature is raised to 350 °C, the crack density is significantly reduced and remains much lower across the entire power range compared to the previous two, indicating that 350 °C preheating can greatly alleviate cracking issues.

**Figure 13:**
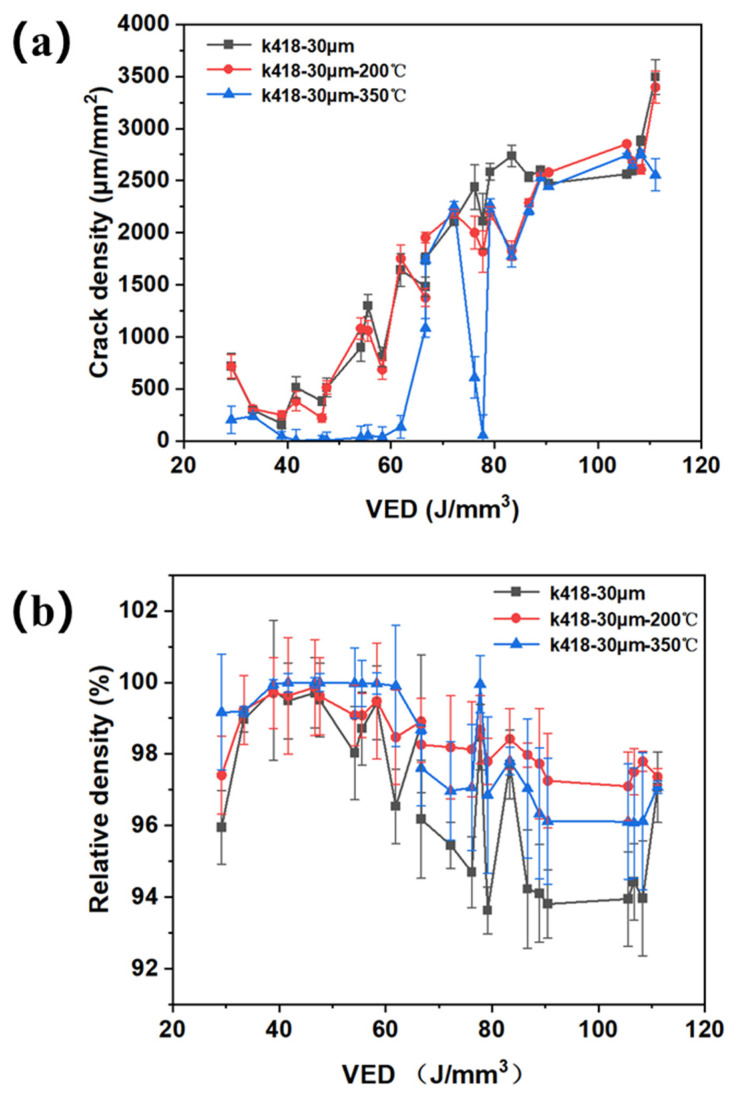
(**a**) The crack density change and laser VED line chart of 30 μm outlining whether the substrate is preheated by SLM processing. (**b**) The relative density change and laser VED line chart of 30 μm outlining whether the substrate is preheated by SLM processing.

The variation in crack density of samples with different substrate preheating temperatures under a layer thickness of 30 μm, along with the VED line graph, is shown in Figure 13a. In the low VED region (20–60 J/mm^3^), crack density increases sharply with increasing VED. In the high VED region (>60 J/mm^3^), the rate of increase in crack density slows down and gradually reaches a high and stable range. The curves for no preheating and preheating at 200 °C almost completely overlap, with crack density remaining very high across the entire VED range (peak close to 2000 μm/mm^2^). Preheating at 200 °C has a limited effect on suppressing solidification cracks in K418 alloy, as this temperature is insufficient to change its solidification thermodynamic conditions [23]. The curve for preheating at 350 °C is consistently at the lowest position, and crack density is significantly suppressed. In the low VED region, its crack density is far lower than both the non-preheated and 200 °C preheated substrates. In the high VED region (100 J/mm^3^), its peak crack density is also significantly lower than that of the non-preheated group. Preheating at 350 °C can greatly reduce crack density and is an effective measure to inhibit cracking. The variation in the relative density of samples with different substrate preheating temperatures under a layer thickness of 30 μm, along with the VED line graph, is shown in Figure 13b. The relative density of all samples increases with increasing VED, which is a general rule in the SLM process. In the low-VED region, relative density increases rapidly with VED; when VED exceeds 60 J/mm^3^, the curve enters a high-density plateau (>98.5%), with the rate of increase slowing down. In the low-to-mid-VED region (<80 J/mm^3^), the relative density of the 200 °C preheated sample is higher than that of the non-preheated sample. This indicates that preheating at 200 °C improves melt flowability and promotes densification. In the high-VED region, both eventually reach an extremely high relative density close to 99%. The 350 °C preheated sample consistently has the highest relative density across the entire VED range. In particular, in the low-VED region (<40 J/mm^3^), its relative density advantage is most pronounced, about 2–3% higher than the non-preheated group. This demonstrates that preheating at 350 °C can significantly reduce the critical energy density required to achieve full densification.

**Figure 14:**
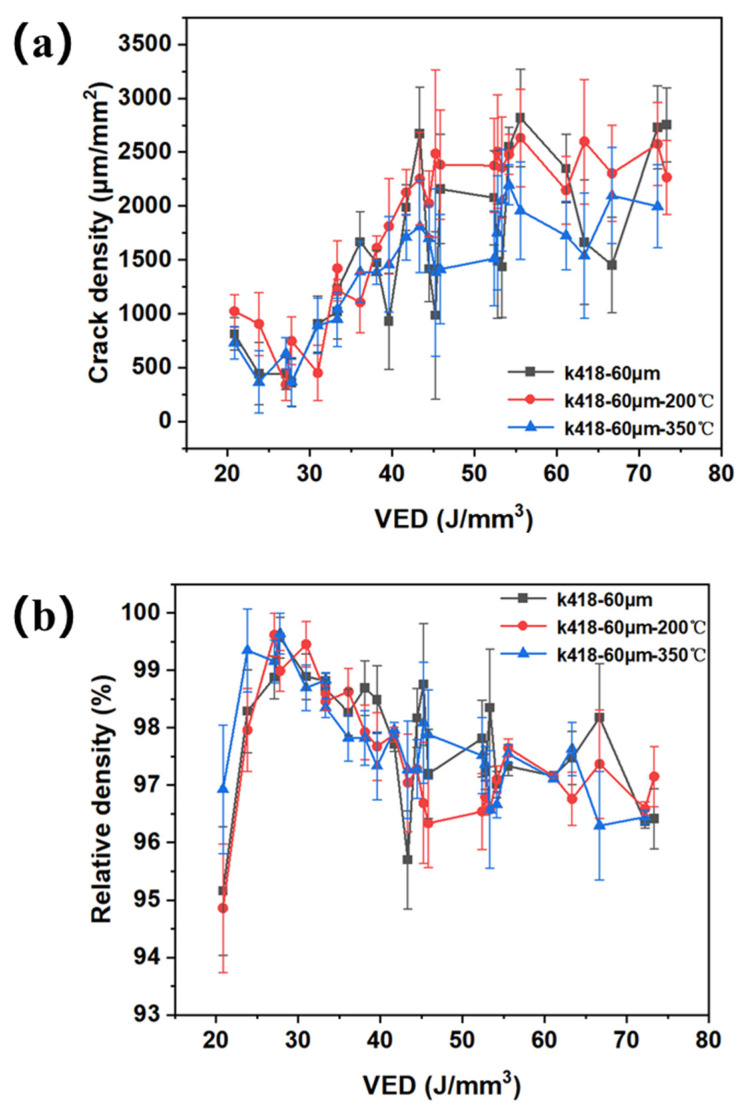
(**a**) The crack density change and laser VED line chart of whether the substrate is preheated by SLM processing. (**b**) The relative density change and laser VED line chart of 60 μm of whether the substrate is preheated by SLM processing.

The crack density changes and VED line plots of samples at different preheating temperatures of the substrate with a layer thickness of 60 μm are shown in Figure 14a. In the lower VED range (20–50 J/mm^3^), the crack density increases rapidly with the increase in VED. In the higher VED range (>50 J/mm^3^), the crack density growth rate slows down and gradually enters a high plateau area. Comparing the substrate without preheating, preheating at 200 °C, and preheating at 350 °C, it is found that the crack density of the substrate is consistent with that under the condition of no preheating. The above results show that it is difficult to reduce cracks when the substrate is preheated under the condition of a layer thickness of 60 μm. The relative density change in the sample and the laser VED line plot are shown in Figure 14b. The relative density of the samples all increased with VED, which is a direct reflection of the energy input promoting melting. In the low-VED region, the relative density gradient is large; after exceeding 50 J/mm^3^, all curves enter the high-density plateau area (>99%), and the densification process tends to be completed. Without warming up, its relative density curve is always at its lowest position. At the low-VED range, the relative density was significantly lower than that of the preheated sample. The 200 °C preheated sample has a curve that is always between no preheating and 350 °C preheating. In the low–mid-VED region, its relative density is systematically higher than that of the unwarmed sample but below the 350 °C preheated sample. This indicates that the preheating energy of 200 °C partially improves melt fluidity and promotes densification. The 350 °C preheating sample, with its curve always at the top, has the highest relative density in the entire VED range. In the low-VED region (<30 J/mm^3^), the relative density advantage is most obvious. This demonstrates that 350 °C preheating significantly reduces the critical energy density required to achieve complete densification, allowing for better melting with less energy.

The above results indicate that preheating to 200 °C can improve melt flowability and increase relative density, but it is insufficient to reduce cooling rates and thermal stress to suppress cracking. Its effect is limited to promoting densification. Preheating to 350 °C reaches a critical temperature. It can significantly improve melt flowability and reduce cooling rates, thereby simultaneously achieving the two effects of promoting densification and suppressing solidification cracks: good melt flowability allows for thorough wetting of the powder and backfilling of shrinkage voids. Lower cooling rates reduce temperature gradients and thermal stress while providing time for elemental diffusion, alleviating harmful grain boundary segregation and enhancing grain boundary strength [24,25,26]. Preheating in the low-VED (volumetric energy density) range is crucial. Preheating to 350 °C can “compensate” for insufficient energy input, achieving high relative density and low crack density even at a lower VED. In the high-VED range, increasing VED can forcibly achieve high relative density (all samples > 98.5%), but it cannot solve the problem of high crack density (samples without preheating and with 200 °C preheating have extremely high crack density). Only preheating to 350 °C can control crack density at a relatively acceptable level in this range. To achieve both high density and low crack sensitivity, a process optimization strategy with a preheating temperature of 350 °C or higher is necessary: preheat to 350 °C and select a medium level of VED (60–80 J/mm^3^). This parameter range is the best balance between “high relative density” and “lower crack density”, allowing for optimal overall formation quality at a lower energy cost. In addition, increasing the layer thickness to 60 μm can somewhat reduce the temperature gradient, thereby lowering the risk of cracking; however, under the 60 μm layer thickness, substrate preheating is ineffective in reducing cracking, mainly due to excessive thermal accumulation promoting material melting, increasing melt pool volume, and causing greater shrinkage during subsequent solidification, which leads to higher stress and an increased risk of cracking.

### 3.3. Characterization of Additive Manufacturing Cracks and Analysis of Cracking Mechanism

**Figure 15:**
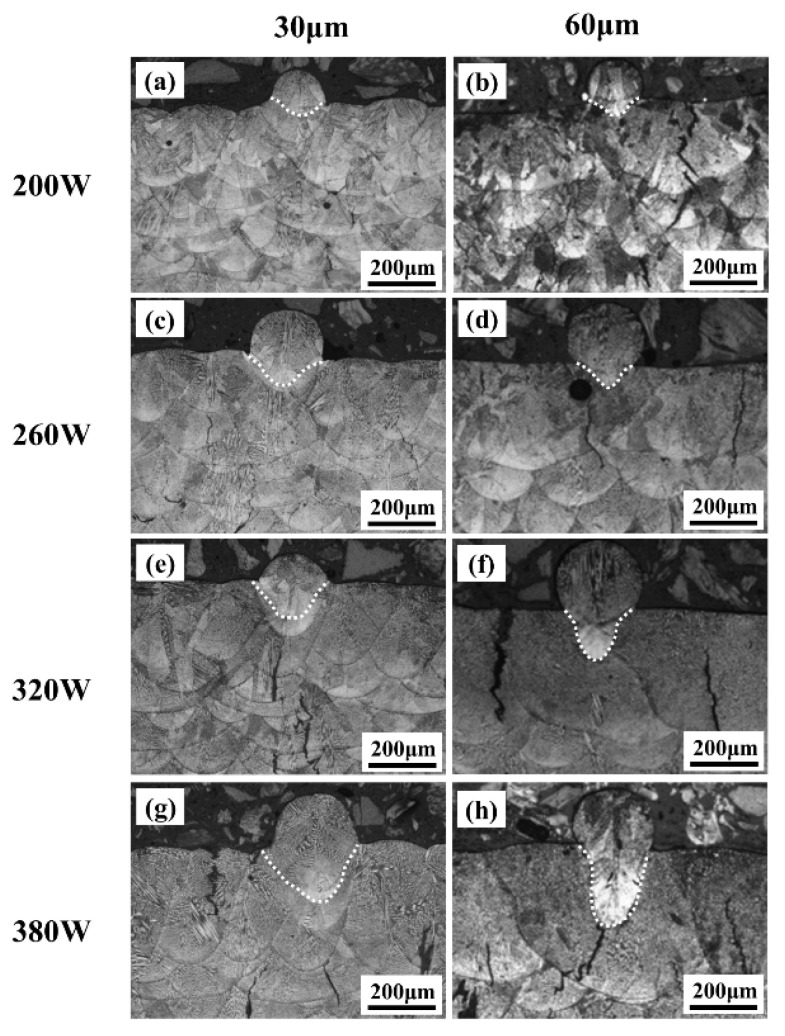
The morphology of K418 weld pool with different layer thicknesses and power at a scanning rate of 1200 mm/s: (**a**) 30 μm layer thickness, power of 200 W; (**b**) 60 μm layer thickness, power of 200 W; (**c**) 30 μm layer thickness, power of 260 W; (**d**) 60 μm layer thickness, power of 260 W; (**e**) 30 μm layer thickness, power of 320 W; (**f**) 60 μm layer thickness, power of 320 W; (**g**) 30 μm layer thickness, power of 380 W; (**h**) 60 μm layer thickness, power of 380 W.

The top molten pool morphology of eight as-printed samples after etching was observed and captured using an optical microscope, as shown in Figure 15. As the power increased from 200 W to 380 W, the morphology of the molten pool changed. At low power (200 W), the molten pool was discontinuous, with obvious lack-of-fusion defects. At medium power (260 W), the molten pool became continuous and stable. At high power (≥320 W), the molten pool was wider and deeper, but defects such as pores began to appear. Under the same power level, a thicker layer helped to form a more stable and continuous melt track, with the most significant effect observed especially at medium power conditions.

**Figure 16:**
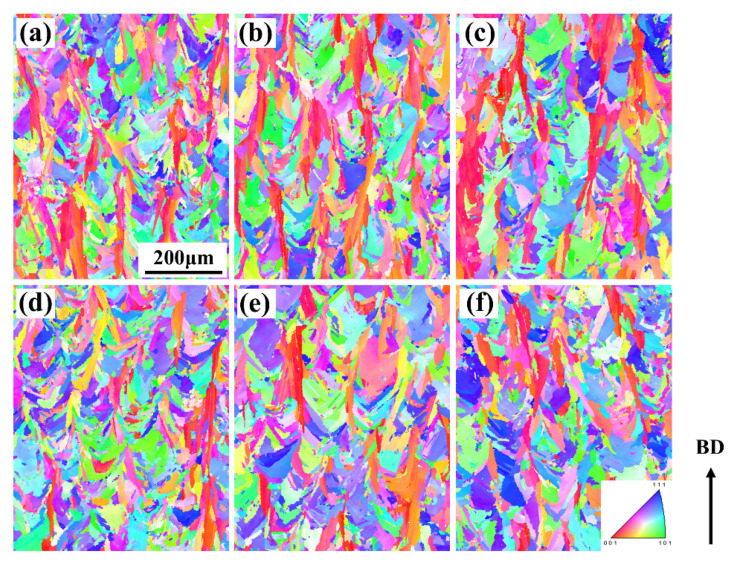
Grain orientation of K418 superalloy with a scanning rate of 1200 mm/s before and after preheating under different layer thicknesses and power: (**a**) 30 μm without preheating, power 200 W; (**b**) 30 μm preheating at 350 °C, power 200 W; (**c**) 30 μm preheating at 350 °C, power 320 W; (**d**) 60 μm without preheating, power 200 W; (**e**) 60 μm preheating at 350 °C, power 200 W; (**f**) 60 μm preheating at 350 °C, power 320 W.

To thoroughly investigate the causes of cracking, advanced EBSD characterization was employed to analyze the grain orientation and grain boundary characteristics in the region near the cracks. The results are shown in Figure 16. Comparing Figure 16a and Figure 16d, it can be observed that under the same low power (200 W) and without preheating, the grains in the 30 μm layer are fine but highly irregular in shape, appearing as elongated and curved columnar grains. This is attributed to the extremely high cooling rate and significant thermal stress induced by the thin layer, causing grain distortion under stress. In contrast, the grains in the 60 μm layer are noticeably coarsened, with straighter and more regular morphology, indicating that the thicker powder layer slows the cooling rate, reduces internal stress, and allows for more sufficient epitaxial growth of the grains.

Comparing Figure 16 with Figure 16b and Figure 16d with Figure 16e, the introduction of 350 °C preheating effectively reduces the temperature gradient and cooling rate, thereby significantly decreasing thermal stress. On the basis of preheating, increasing the power from 200 W to 320 W shows no obvious change in grain size. The higher energy input provides more heat, lowers the cooling rate, and promotes grain growth.

**Figure 17:**
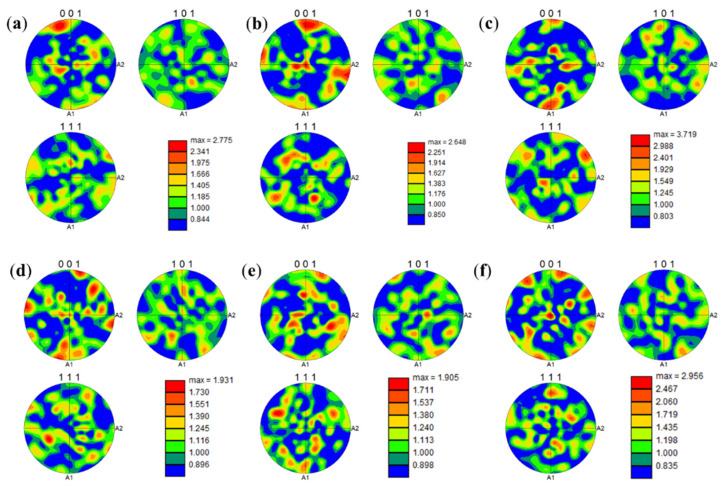
Electrode diagrams of K418 superalloy with scanning rates of 1200 mm/s before and after preheating under different layer thicknesses and powers: (**a**) 30 μm without preheating, power 200 W; (**b**) 30 μm preheating at 350 °C, power 200 W; (**c**) 30 μm preheating at 350 °C, power 320 W; (**d**) 30 μm without preheating, power 200 W; (**e**) 30 μm preheating at 350 °C, power 200 W; (**f**) 30 μm preheating at 350 °C, power 320 W.

The maximum intensity value in the pole figure directly reflects the strength of the texture, with higher values indicating a stronger preferred orientation. The maximum pole figure intensities for the samples with a 30 μm layer thickness (Figure 17a–c: 2.775, 2.648, 3.719) are significantly higher than those for the samples with a 60 μm layer thickness (Figure 17d–f: 1.931, 1.905, 2.956). This indicates that a smaller layer thickness combined with higher energy input (320 W) significantly strengthens the <001> texture. The underlying mechanism is that the combination of a smaller layer thickness and higher power creates an extremely steep temperature gradient, promoting epitaxial grain growth along the direction of maximum heat flow (i.e., the BD), thereby strengthening the cube texture. Preheating reduces the temperature gradient and, to some extent, disrupts the epitaxial growth of grains. Thus, by comparing Figure 17a and Figure 17b as well as Figure 17d and Figure 17e, it can be observed that the texture intensity shows a slight weakening trend after preheating.

**Figure 18:**
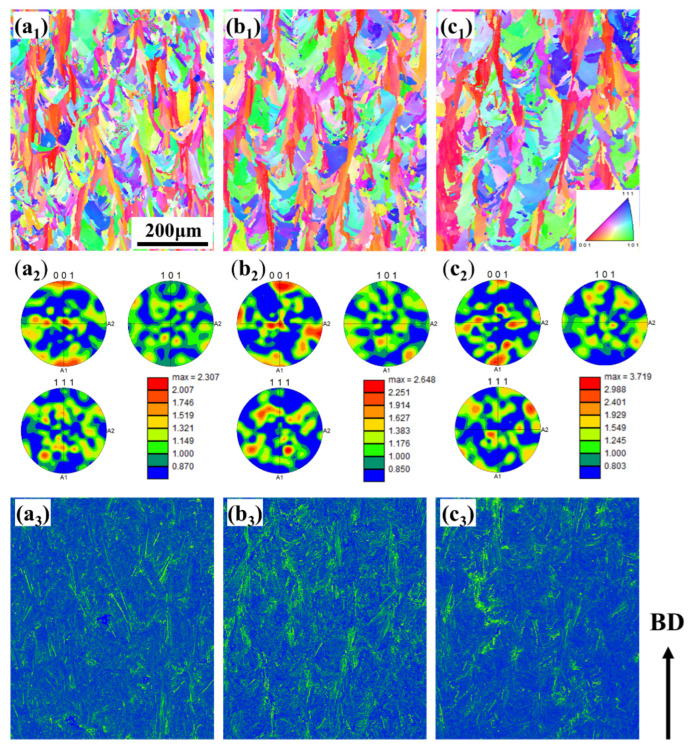
Preheated 350 °C sample with layer thickness of 30 μm and scanning rate of 1200 mm/s K418 superalloy EBSD diagram: (**a_1_**–**a_3_**) 140 W, 200 W, 320 W; (**b_1_**–**b_3_**) 140 W, 200 W, 320 W; (**c_1_**–**c_3_**) 140 W, 200 W, 320 W.

Figure 18 shows EBSD maps of K418 superalloy printed with a 350 °C preheating temperature, 30 μm layer thickness, and a scanning speed of 1200 mm/s under different laser powers. At the lower power of 140 W, the grains are the finest but irregular in shape, exhibiting elongated columnar grains and some equiaxed grains. This indicates that the low energy input results in a small melt pool and an extremely high cooling rate, while the substantial thermal stress likely causes grain bending and deformation. The KAM map reveals highly concentrated micro-strain and an extremely high dislocation density. This is because the very high cooling rate at low power generates significant thermal stress, and the fine grain boundaries and substructures hinder dislocation motion, leading to dislocation tangles and pile-ups. The texture strength is relatively weak. The rapid and unstable solidification process, along with a high nucleation rate, suppress strong preferred growth. When the power is increased to 200 W, the grain size increases, and the columnar grains become coarser, showing typical epitaxial growth characteristics. This suggests that the increased power provides a more stable and wider melt pool, allowing grains to grow sufficiently along the direction of maximum heat flow (i.e., the building direction, BD). A significant enhancement in texture strength is observed. A pronounced preferred orientation of grains along the <001> direction becomes evident, which is the most common texture in nickel-based alloys during SLM, originating from the epitaxial competitive growth of grains with <001> orientation at the bottom of the melt pool. The KAM map shows a decrease in micro-strain and dislocation density. The slower cooling rate allows some dislocations to rearrange and annihilate through recovery processes, thereby relaxing part of the residual stress.

At 320 W, the grains coarsen further, forming very coarse columnar grains that almost span the entire displayed area. The extremely high energy input creates a large and deep melt pool, and the relatively reduced cooling rate provides conditions for sufficient grain growth. The texture strength reaches its maximum at 3.719, showing a strong cube texture (<001>//BD). This indicates that at high power, the orientation selection and competitive growth of grains proceed most thoroughly. The KAM values decrease further, with the map dominated by blue and green colors, indicating the lowest levels of micro-strain and dislocation density. The high-power condition provides the most sufficient slow cooling, offering the optimal thermodynamic conditions for stress relief and dislocation reorganization. However, special attention should be paid to the KAM values at grain boundaries, as the boundaries of coarse columnar grains are often dangerous sites for dislocation accumulation and crack initiation. Laser power, by controlling the melt pool size and thermal gradient, dominates the grain growth behavior. Increased power → increased energy input → larger melt pool, decreased cooling rate → sufficient epitaxial grain growth → grain coarsening and formation of a strong <001> texture. Laser power influences residual stress and dislocation density by altering the cooling rate. Increased power → decreased cooling rate → reduced thermal stress, while dislocations have more time for recovery and reorganization → significant reduction in micro-strain (dislocation density).

Under the conditions of 350 °C preheating and a 30 μm layer thickness, laser power has a decisive influence on the microstructure of SLM-fabricated K418 alloy: as the laser power increases from 140 W to 320 W, the grains coarsen significantly, and the strength of the <001> texture continuously strengthens. This indicates that higher power is more favorable for epitaxial growth and orientation competition of grains. With increasing laser power, the micro-strain and dislocation density decrease significantly. This shows that higher power, by slowing the cooling rate, effectively promotes stress relief and dislocation recovery, which helps reduce cracking tendency. Although the high power (320 W) results in the lowest dislocation density and the coarsest grains, its intense texture may lead to anisotropy in mechanical properties. In contrast, the medium power (200 W) may maintain better performance uniformity while achieving relatively coarse grains and lower dislocation density. Therefore, the selection of power requires a balance between “low stress” and “isotropic properties.” Subsequent mechanical property tests (such as room-temperature and high-temperature tensile tests; fatigue performance) should be conducted to determine the optimal power.

**Figure 19:**
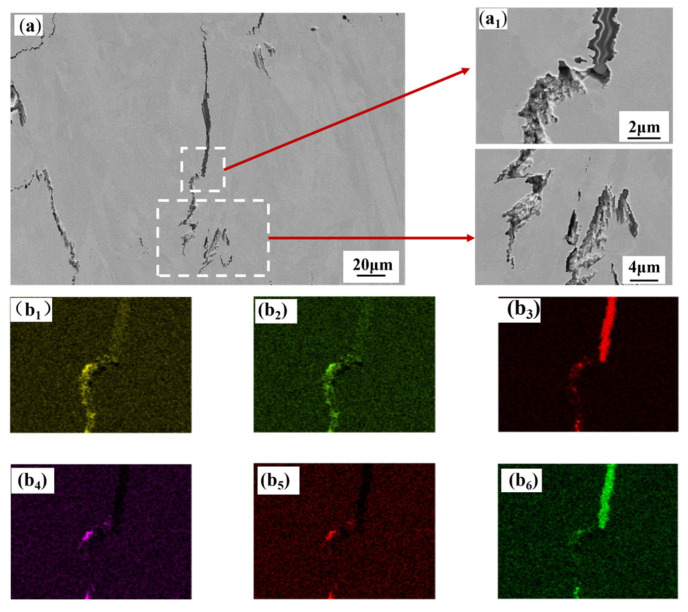
The SEM and its corresponding EDS diagram of the liquefied crack of the printed K418 sample prepared by SLM. (**a**) a crack extracted from the sample, (**a_1_**) which is a magnified view of the white area in (**a**). (**b_1_**) O, (**b_2_**) Cr, (**b_3_**) C, (**b_4_**) Si, (**b_5_**) W, (**b_6_**) B.

**Figure 20:**
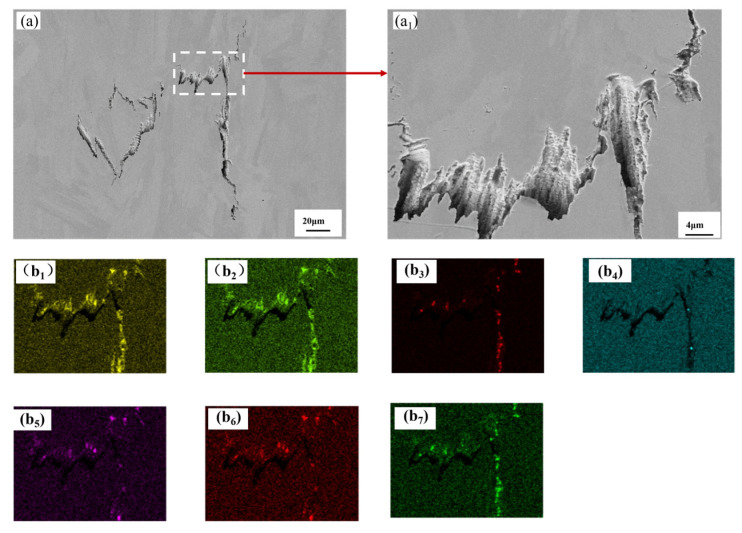
The SEM and its corresponding EDS diagram of the solidified crack of the printed K418 sample prepared by SLM. (**a**) a crack extracted from the sample, (**a_1_**) which is a magnified view of the white area in (**a**). (**b_1_**) O, (**b_2_**) Cr, (**b_3_**) C, (**b_4_**) Al, (**b_5_**) Si, (**b_6_**) W, (**b_7_**) B.

EDS analysis of both cracks clearly shows strong oxygen signals at the crack openings and in the adjacent areas. This indicates that the cracks interacted with oxygen from the environment or the powder surface during or after their formation at high temperatures, leading to high-temperature oxidation. Oxidation can cause grain boundary embrittlement, reduce grain boundary cohesion, and thus provide a preferential path for crack initiation and propagation. Figure 19 and Figure 20 both clearly indicate the segregation of boron at the crack sites. Boron is a common grain-boundary-strengthening element in nickel-based superalloys such as K418; however, excessive boron or rapid solidification conditions can easily promote the formation of low-melting-point boride eutectics (e.g., M_3_B_2_ type). These low-melting-point phases can remelt during subsequent thermal cycles of the SLM process, significantly reducing the high-temperature strength and ductility of grain boundaries and making them highly susceptible to liquation cracking or intergranular cracking under substantial thermal stress. In terms of the distribution characteristics of other elements, major alloying elements such as Cr and W show no obvious enrichment at the cracks, and their signals mainly originate from the matrix, indicating that the cracks are not directly caused by compounds of these elements. Si and Al exhibit slight enrichment in certain regions; they may also form low-melting-point eutectic phases (e.g., Ni–Si, Ni–Al), which together with B and O, can exacerbate grain boundary embrittlement.

Based on the above analysis, it is concluded that the cracks in SLM-fabricated K418 alloy are primarily hot cracks propagating along grain boundaries. This is primarily triggered by grain boundary liquation and embrittlement induced by multicomponent eutectic reactions, with boron segregation and oxidation identified as two key contributing factors. The extremely high temperature gradient and rapid cooling rate promote the severe segregation of elements such as boron and oxygen at the last-solidifying grain boundaries, forming a continuous brittle interfacial network, which readily fractures under the thermal stresses generated during the fabrication process.

## 4. Conclusions

This study investigated the effects of laser parameters, layer thickness, and substrate preheating on solidification cracking in LPBF-processed K418 alloy. The main findings are as follows:

(1) Laser power and scanning speed significantly influence defect formation. A moderate volumetric energy density (VED ≤ 60 J/mm^3^) reduces cracking, while higher VED (≥75 J/mm^3^) increases crack susceptibility and insufficient energy leads to lack-of-fusion defects.
(2) For a 30 μm layer, substrate preheating effectively reduces cracking. Preheating to 350 °C, combined with appropriate laser parameters, nearly eliminates cracks by lowering thermal stress and temperature gradients, whereas 200 °C preheating shows limited effectiveness.
(3) Without preheating, a 60 μm layer reduces cracking compared to 30 μm and widens the process window. However, preheating is ineffective at this thickness due to excessive heat accumulation, which enlarges the melt pool and increases shrinkage stress.
(4) Cracks are predominantly hot cracks that propagate along high-angle grain boundaries, where low-melting-point elements (O, B, Si, C) segregate, linking crack formation to grain boundary weakening and solidification stress.
(5) The optimized process parameters are 30 μm layer thickness, 350 °C substrate preheating, and VED = 60–80 J/mm^3^. This combination achieves >99% relative density while minimizing crack density.

## Figures and Tables

**Table 1:** Chemical composition of K418 powder (wt.%).

Element	Ni	Cr	Al	Mo	Nb	Ti	Fe	C	Zr	Si	B	Mn	P
**Content (wt%)**	Bal	12.65	6.03	4.37	2.15	0.81	0.017	0.15	0.12	0.014	0.019	0.014	0.004

**Table 2:** Printing parameters selected in this study.

N	Layer Thickness (μm)	Scanning Speed (mm/s)	Power (W)	Scan Spacing (mm)	Substrate Heating Temperature (°C)	Scanning Strategy
T1	30	600, 800, 1000, 1200, 1400	140, 200, 260, 320, 380	0.1	25	67° alternating
T2	30	600, 800, 1000, 1200, 1400	140, 200, 260, 320, 380	0.1	350	67° alternating
T3	60	600, 800, 1000, 1200, 1400	140, 200, 260, 320, 380	0.1	25	67° alternating
T4	60	600, 800, 1000, 1200, 1400	140, 200, 260, 320, 380	0.1	350	67° alternating

## Data Availability

The original contributions presented in this study are included in the article. Further inquiries can be directed to the corresponding author.
